# Supplementary material for: Mental disorders and intimate partner violence perpetrated by men towards women: A Swedish population-based longitudinal study
Source: PLoS Med. 2019 Dec 17;16(12):e1002995. doi: 10.1371/journal.pmed.1002995 (PMC6917212; doi:10.1371/journal.pmed.1002995)
Supplement: S1 Table — (DOCX) [file pmed.1002995.s002.docx]

S1 Table. Descriptive data for risk factors in unaffected full siblings and matched general population controls

|  |  | Unaffected full siblings | General population controls |
| --- | --- | --- | --- |
| Schizophrenia-spectrum disorders | Sample size | 11,592 | 230,686 |
|  | Follow-up start age^a^ | 31.0 (9.5 SD) | 30.9 (9.5) |
|  | Low income^b^ | 3,975 (36.5%) | 62,519 (28.8%) |
|  | Single status^b^ | 9,085 (79.6%) |  |
|  | Born abroad^b^ | 1,263 (10.9%) | 10,032 (4.3%) |
|  | Previous IPV^b^ | 62 (0.5%) | 580 (0.3%) |
| Bipolar disorder | Sample size | 5,758 | 114,284 |
|  | Follow-up start age | 33.0 (10.2) | 32.9 (10.2 SD) |
|  | Low income | 1,777 (32.7%) | 29,610 (27.4%) |
|  | Single status | 4,230 (75.0%) |  |
|  | Born abroad | 230 (4.0%) | 5,046 (4.4%) |
|  | Previous IPV | 25 (0.4%) | 383 (0.3%) |
| Depressive disorder | Sample size | 36,453 | 718,616 |
|  | Follow-up start age | 30.7 (10.9) | 30.7 (10.9 SD) |
|  | Low income | 10,424 (31.2%) | 178,474 (27.2%) |
|  | Single status | 28,006 (77.9%) |  |
|  | Born abroad | 2,066 (5.7%) | 29,364 (4.1%) |
|  | Previous IPV | 165 (0.5%) | 1,945 (0.3%) |
| Anxiety disorder | Sample size | 28,962 | 575,154 |
|  | Follow-up start age | 29.5 (10.7) | 29.4 (10.7 SD) |
|  | Low income | 8,417 (32.2%) | 146,602 (28.4%) |
|  | Single status | 22,906 (80.2%) | 112,714 (20.0%) |
|  | Born abroad | 1,985 (6.9%) | 24,385 (4.2%) |
|  | Previous IPV | 134 (0.5%) | 1,503 (0.3%) |
| Alcohol use disorder | Sample size | 37,885 | 752,833 |
|  | Follow-up start age | 29.4 (11.2) | 29.4 (11.1 SD) |
|  | Low income | 10,431 (30.9%) | 185,960 (27.8%) |
|  | Single status | 30,531 (81.5%) | 148,394 (20.0%) |
|  | Born abroad | 2,251 (5.9%) | 32,037 (4.3%) |
|  | Previous IPV | 149 (0.4%) | 1,667 (0.2%) |
| Drug use disorder | Sample size | 24,116 | 479,438 |
|  | Follow-up start age | 29.6 (10.5) | 29.5 (10.5 SD) |
|  | Low income | 7,845 (35.3%) | 128,376 (29.2%) |
|  | Single status | 19,463 (81.8%) | 92,856 (19.7%) |
|  | Born abroad | 2,302 (9.5%) | 20,530 (4.3%) |
|  | Previous IPV | 117 (0.5%) | 1,138 (0.2%) |
| ADHD | Sample size | 22,576 | 449,150 |
|  | Follow-up start age | 24.1 (11.2) | 24.1 (11.2 SD) |
|  | Low income | 6,603 (31.6%) | 113,289 (27.3%) |
|  | Single status | 19,795 (88.7%) | 52,784 (12.0%) |
|  | Born abroad | 965 (4.3%) | 17,180 (3.8%) |
|  | Previous IPV | 96 (0.4%) | 864 (0.2%) |

Note. SD = standard deviation. IPV = intimate partner violence. ADHD = attention deficit hyperactivity disorder. ^a^ figures represent mean (standard deviation). ^b^ figures represent N(%).
