# Supplementary material for: Mental disorders and intimate partner violence perpetrated by men towards women: A Swedish population-based longitudinal study
Source: PLoS Med. 2019 Dec 17;16(12):e1002995. doi: 10.1371/journal.pmed.1002995 (PMC6917212; doi:10.1371/journal.pmed.1002995)
Supplement: S2 Table — (DOCX) [file pmed.1002995.s003.docx]

S2 Table. Hazard ratio (HR) for confounders in the models comparing risk of intimate partner violence against women in men with mental disorders with their matched general population controls

|  | Individuals with mental disorders | | | | | |
| --- | --- | --- | --- | --- | --- | --- |
|  | Family low-income | | Single status | | Immigrant status | |
|  | HR | (CI) | HR | (CI) | HR | (CI) |
| Schizophrenia-spectrum disorders | 2.0 | 1.8-2.2 | 0.9 | 0.8-1.0 | 3.5 | 3.2-3.9 |
| Bipolar disorder | 2.3 | 1.9-2.7 | 1.1 | 0.9-1.3 | 4.4 | 3.7-5.2 |
| Depressive disorder | 2.4 | 2.2-2.5 | 1.2 | 1.1-1.3 | 4.3 | 4.0-4.6 |
| Anxiety disorder | 2.3 | 2.1-2.5 | 1.3 | 1.1-1.4 | 4.7 | 4.3-5.1 |
| Alcohol use disorder | 2.1 | 1.9-2.2 | 1.2 | 1.1-1.3 | 4.4 | 4.1-4.7 |
| Drug use disorder | 1.7 | 1.6-1.9 | 1.2 | 1.1-1.3 | 4.1 | 3.8-4.4 |
| ADHD | 2.2 | 2.0-2.5 | 1.5 | 1.2-1.8 | 6.1 | 5.4-7.0 |
| Autism | 2.1 | 1.5-2.9 | 2.1 | 1.3-3.5 | 6.4 | 4.7-8.7 |
| Personality disorders | 2.0 | 1.8-2.2 | 1.2 | 1.0-1.3 | 3.8 | 3.4-4.3 |

Note. HR = hazard ratios. CI = confidence interval. ADHD = attention deficit hyperactivity disorder.
