# Supplementary material for: Mental disorders and intimate partner violence perpetrated by men towards women: A Swedish population-based longitudinal study
Source: PLoS Med. 2019 Dec 17;16(12):e1002995. doi: 10.1371/journal.pmed.1002995 (PMC6917212; doi:10.1371/journal.pmed.1002995)
Supplement: S3 Table — (DOCX) [file pmed.1002995.s004.docx]

S3 Table. Crude hazard ratio (cHR) of intimate partner violence against women in men with mental disorders by psychiatric comorbidity

|  | Comorbidity  of alcohol use disorder | | | |  | Comorbidity of drug use disorder | | | |  | Comorbidity of personality disorder | | | |
| --- | --- | --- | --- | --- | --- | --- | --- | --- | --- | --- | --- | --- | --- | --- |
|  | Yes | | No | |  | Yes | | No | |  | Yes | | No | |
|  | cHR | (CI) | cHR | (CI) |  | cHR | (CI) | cHR | (CI) |  | cHR | (CI) | cHR | (CI) |
| Schizophrenia-spectrum disorders | 3.8 | 3.0 - 4.8 | 1.5 | 1.2 - 1.8 |  | 3.9 | 3.2 - 4.8 | 1.3 | 1.1 - 1.6 |  | 4.1 | 3.2 - 5.2 | 1.5 | 1.2 - 1.8 |
| Bipolar disorder | 5.3 | 3.7 - 7.7 | 1.3 | 0.9 - 1.8 |  | 5.2 | 3.5 - 7.8 | 1.5 | 1.1 - 2.1 |  | 4.2 | 2.4 - 7.2 | 1.9 | 1.4 - 2.5 |
| Depressive disorder | 7.2 | 6.3 - 8.2 | 2.3 | 2.1 - 2.6 |  | 6.8 | 5.9 - 7.9 | 2.6 | 2.4 - 2.9 |  | 5.3 | 4.4 - 6.5 | 3.1 | 2.8 - 3.3 |
| Anxiety disorder | 6.9 | 5.7 - 8.4 | 1.8 | 1.6 - 2.1 |  | 7.7 | 6.4 - 9.3 | 1.7 | 1.5 - 2.0 |  | 5.1 | 3.8 - 6.9 | 2.3 | 2.1 - 2.6 |
| Alcohol use disorder |  |  |  |  |  | 9.3 | 8.5 - 10.3 | 4.8 | 4.4 - 5.2 |  | 9.7 | 8.2 - 11.4 | 5.8 | 5.4 - 6.1 |
| Drug use disorder | 9.3 | 8.5 - 10.2 | 5.7 | 5.2 - 6.2 |  |  |  |  |  |  | 9.2 | 8.0 - 10.5 | 6.7 | 6.3 - 7.3 |
| ADHD | 9.3 | 7.6 - 11.5 | 3.8 | 3.2 - 4.5 |  | 8.4 | 7.0 - 10.1 | 3.5 | 2.9 - 4.2 |  | 10.5 | 8.1 - 13.5 | 4.1 | 3.6 - 4.8 |
| Autism | 4.1 | 1.2 - 14.2 | 0.3 | 0.1 - 0.9 |  | 4.5 | 1.3 - 15.5 | 0.3 | 0.1 - 0.9 |  | 3.1 | 0.7 - 13.9 | 0.4 | 0.1 - 1.0 |
| Personality disorders | 7.4 | 6.2 - 8.9 | 3.5 | 3.0 - 4.2 |  | 6.9 | 5.9 - 8.1 | 3.3 | 2.8 - 4.0 |  |  |  |  |  |

Note. cHR = crude hazard ratio (not adjusted for any covariates). CI = confidence interval. ADHD = attention deficit hyperactivity disorder.
