# Supplementary material for: Mental disorders and intimate partner violence perpetrated by men towards women: A Swedish population-based longitudinal study
Source: PLoS Med. 2019 Dec 17;16(12):e1002995. doi: 10.1371/journal.pmed.1002995 (PMC6917212; doi:10.1371/journal.pmed.1002995)
Supplement: S4 Table — (DOCX) [file pmed.1002995.s005.docx]

S4 Table. Hazard ratio (HR) and ratio of hazard ratios (RHR) of general domestic violence in men with mental disorders and their unaffected full siblings.

|  | Individuals with mental disorders | | | |  |  | Unaffected full siblings | | | |  |  | | |
| --- | --- | --- | --- | --- | --- | --- | --- | --- | --- | --- | --- | --- | --- | --- |
|  | cHR | (CI) | aHR | (CI) | *p* |  | cHR | (CI) | aHR | (CI) | *p* | RHR | (CI) | *p* |
| Schizophrenia-spectrum disorders | 2.1 | 1.9 - 2.4 | 1.6 | 1.4-1.9 | <.001 |  | 2.5 | 2.1 - 3.1 | 2.1 | 1.7-2.5 | <.001 | 0.8 | 0.6-1.0 | .03 |
| Bipolar disorder | 2.2 | 1.8 - 2.8 | 2.2 | 1.8-2.8 | <.001 |  | 1.4 | 0.9 - 2.3 | 1.4 | 0.8-2.3 | .21 | 1.6 | 0.9-2.8 | .12 |
| Depressive disorder | 3.1 | 2.9 - 3.3 | 2.7 | 2.5-2.9 | <.001 |  | 1.3 | 1.1 - 1.6 | 1.2 | 1.0-1.5 | .03 | 2.3 | 1.8-2.8 | <.001 |
| Anxiety disorder | 2.4 | 2.2 - 2.7 | 2.3 | 2.1-2.6 | <.001 |  | 1.8 | 1.5 - 2.2 | 1.6 | 1.3-2.0 | <.001 | 1.4 | 1.1-1.8 | <.001 |
| Alcohol use disorder | 5.4 | 5.1 - 5.7 | 6.2 | 5.9-6.6 | <.001 |  | 1.8 | 1.5 - 2.1 | 1.7 | 1.4-2.0 | <.001 | 3.7 | 3.0-4.4 | <.001 |
| Drug use disorder | 6.6 | 6.2 - 7.0 | 7.0 | 6.6-7.5 | <.001 |  | 2.3 | 2.0 - 2.8 | 2.0 | 1.7-2.4 | <.001 | 3.5 | 2.9-4.2 | <.001 |
| ADHD | 4.5 | 4.1 - 5.1 | 5.6 | 5.0-6.4 | <.001 |  | 2.0 | 1.5 - 2.6 | 2.0 | 1.5-2.6 | <.001 | 2.8 | 2.1-3.8 | <.001 |
| Autism | 0.8 | 0.5 - 1.4 | 0.9 | 0.5-1.7 | .82 |  | 2.0 | 1.2 - 3.3 | 1.7 | 1.0-3.0 | .07 | 0.5 | 0.2-1.2 | .13 |
| Personality disorders | 4.6 | 4.2 - 5.2 | 4.2 | 3.7-4.7 | <.001 |  | 2.9 | 2.3 - 3.7 | 2.6 | 2.1-3.2 | <.001 | 1.6 | 1.3-2.1 | <.001 |

Note. General domestic violence includes threats, violence, and sexual assaults against a person that offender has or has had a close relationship to, including partners, children, parent, sibling of the offender (crime codes: 0411, 0412, 0422, 0423, 0424, 0425, 0440, 0441, 0442, and 0443). cHR = crude hazard ratio (not adjusted for any covariates). CI = confidence interval. aHRs = adjusted hazard ratios. RHR = ratio of hazard ratios. ADHD = attention deficit hyperactivity disorder. Both individuals with mental disorders and their unaffected full siblings were compared with 20 age- and gender- matched general population controls. aHR analyses were adjusted for family income, single status, and immigrant status.
