# Supplementary material for: Mental disorders and intimate partner violence perpetrated by men towards women: A Swedish population-based longitudinal study
Source: PLoS Med. 2019 Dec 17;16(12):e1002995. doi: 10.1371/journal.pmed.1002995 (PMC6917212; doi:10.1371/journal.pmed.1002995)
Supplement: S5 Table — (DOCX) [file pmed.1002995.s006.docx]

S5 Table. Hazard ratio (HR) of intimate partner violence against women in men with an inpatient and an outpatient diagnosis of mental disorders

|  | Individuals with  an inpatient diagnosis | | | | |  |  | Individuals with  an outpatient diagnosis | | | | | |
| --- | --- | --- | --- | --- | --- | --- | --- | --- | --- | --- | --- | --- | --- |
|  | n | cHR | (CI) | aHR | (CI) | *p* |  | n | cHR | (CI) | aHR | (CI) | *p* |
| Schizophrenia-spectrum disorders | 14,634 | 2.0 | 1.7 - 2.3 | 1.5 | 1.3-1.8 | <.001 |  | 11,451 | 1.9 | 1.5 - 2.5 | 1.3 | 1.0-1.7 | .05 |
| Bipolar disorder | 2,721 | 1.8 | 1.1 - 2.9 | 1.7 | 1.1-2.8 | .02 |  | 9,344 | 2.4 | 1.8 - 3.2 | 2.4 | 1.8-3.3 | <.001 |
| Depressive disorder | 17,538 | 4.3 | 3.7 - 4.9 | 3.9 | 3.4-4.5 | <.001 |  | 70,644 | 3.0 | 2.7 - 3.3 | 2.5 | 2.3-2.8 | <.001 |
| Anxiety disorder | 6,617 | 3.4 | 2.7 - 4.4 | 3.5 | 2.7-4.5 | <.001 |  | 53,738 | 2.4 | 2.1 - 2.7 | 2.3 | 2.0-2.6 | .003 |
| Alcohol use disorder | 44,636 | 6.2 | 5.7 - 6.7 | 7.3 | 6.7-7.9 | <.001 |  | 38,095 | 6.0 | 5.5 - 6.7 | 6.6 | 5.9-7.4 | <.001 |
| Drug use disorder | 28,864 | 7.7 | 7.1 - 8.3 | 8.6 | 7.8-9.4 | <.001 |  | 29,037 | 6.3 | 5.6 - 7.0 | 6.3 | 5.5-7.2 | <.001 |
| ADHD | 3,731 | 5.5 | 4.0 - 7.5 | 6.4 | 4.5-9.2 | <.001 |  | 45,596 | 5.0 | 4.3 - 5.7 | 6.5 | 5.5-7.6 | <.001 |
| Autism | 1,445 | 0.7 | 0.2 - 2.9 | 0.9 | 0.2-4.2 | .93 |  | 8,084 | 0.5 | 0.2 - 1.3 | 0.6 | 0.2-1.7 | .33 |
| Personality disorders | 6,588 | 5.4 | 4.6 - 6.4 | 5.1 | 4.2-6.2 | <.001 |  | 13,262 | 4.3 | 3.6 - 5.1 | 3.6 | 3.0-4.4 | <.001 |

Note. cHR = crude hazard ratio (not adjusted for any covariates). CI = confidence interval. aHRs = adjusted hazard ratios. ADHD = attention deficit hyperactivity disorder. Individuals with an inpatient or outpatient diagnosis of mental disorders were compared with general population controls and matched by age. aHR analyses were adjusted for family income, single status, and immigrant status.
