# Supplementary material for: Mental disorders and intimate partner violence perpetrated by men towards women: A Swedish population-based longitudinal study
Source: PLoS Med. 2019 Dec 17;16(12):e1002995. doi: 10.1371/journal.pmed.1002995 (PMC6917212; doi:10.1371/journal.pmed.1002995)
Supplement: S6 Table — (DOCX) [file pmed.1002995.s007.docx]

S6 Table. Hazard ratio (HR) and ratio of hazard ratios (RHR) of intimate partner violence against women in men with mental disorders and their unaffected full siblings after excluding individuals with a previous IPV

|  | Individuals with mental disorders | | | | | |  | Unaffected full siblings | | | | | |  | | |
| --- | --- | --- | --- | --- | --- | --- | --- | --- | --- | --- | --- | --- | --- | --- | --- | --- |
|  | n | cHR | (CI) | aHR | (CI) | *p* |  | n | cHR | (CI) | aHR | (CI) | *p* | RHR | (CI) | *p* |
| Schizophrenia-spectrum disorders | 25,713 | 2.0 | 1.7 - 2.2 | 1.5 | 1.3-1.7 | <.001 |  | 11,530 | 2.7 | 2.2 - 3.4 | 2.2 | 1.8-2.7 | <.001 | 0.7 | 0.5-0.9 | <.001 |
| Bipolar disorder | 11,867 | 2.2 | 1.7 - 2.8 | 2.2 | 1.7-2.8 | <.001 |  | 5,733 | 1.2 | 0.7 - 2.3 | 1.2 | 0.6-2.2 | .61 | 1.8 | 0.9-3.7 | .09 |
| Depressive disorder | 86,848 | 3.4 | 3.1 - 3.6 | 2.9 | 2.7-3.2 | <.001 |  | 36,288 | 1.3 | 1.1 - 1.7 | 1.2 | 0.9-1.5 | .14 | 2.4 | 1.9-3.2 | <.001 |
| Anxiety disorder | 59,565 | 2.5 | 2.3 - 2.8 | 2.5 | 2.2-2.8 | <.001 |  | 28,828 | 1.7 | 1.4 - 2.2 | 1.5 | 1.2-1.9 | <.001 | 1.7 | 1.3-2.1 | <.001 |
| Alcohol use disorder | 80,987 | 6.2 | 5.8 - 6.6 | 7.2 | 6.7-7.7 | <.001 |  | 37,736 | 1.7 | 1.4 - 2.0 | 1.6 | 1.4-1.9 | <.001 | 4.5 | 3.8-5.3 | <.001 |
| Drug use disorder | 56,541 | 7.3 | 6.9 - 7.8 | 7.9 | 7.3-8.5 | <.001 |  | 23,999 | 2.4 | 2.0 - 2.9 | 2.1 | 1.7-2.5 | <.001 | 3.8 | 3.1-4.6 | <.001 |
| ADHD | 48,523 | 5.2 | 4.5 - 5.9 | 6.6 | 5.7-7.7 | <.001 |  | 22,480 | 2.1 | 1.5 - 2.9 | 2.1 | 1.6-2.8 | <.001 | 3.1 | 2.3-4.3 | <.001 |
| Autism | 9,511 | 0.5 | 0.2 - 1.2 | 0.7 | 0.3-1.6 | .39 |  | 4,809 | 2.2 | 1.2 - 4.0 | 2.2 | 1.2-3.9 | .01 | 0.3 | 0.1-0.9 | .03 |
| Personality disorders | 19,203 | 4.9 | 4.4 - 5.5 | 4.4 | 3.9-5.1 | <.001 |  | 9,066 | 3.0 | 2.3 - 3.9 | 2.5 | 2.0-3.3 | <.001 | 1.8 | 1.3-2.3 | <.001 |

Note. cHR = crude hazard ratio (not adjusted for any covariates). CI = confidence interval. aHRs = adjusted hazard ratios. RHR = ratio of hazard ratios. ADHD = attention deficit hyperactivity disorder. Both individuals with mental disorders and their unaffected full siblings were compared with 20 age- and gender- matched general population controls. aHR analyses were adjusted for family income, single status, and immigrant status.
