# Supplementary material for: Mental disorders and intimate partner violence perpetrated by men towards women: A Swedish population-based longitudinal study
Source: PLoS Med. 2019 Dec 17;16(12):e1002995. doi: 10.1371/journal.pmed.1002995 (PMC6917212; doi:10.1371/journal.pmed.1002995)
Supplement: S7 Table — (DOCX) [file pmed.1002995.s008.docx]

S7 Table. Adjusted hazard ratio (aHR) and ratio of hazard ratios (RHR) of intimate partner violence against women in men with mental disorders after adjustment of prior alcohol and drug use disorders

|  | Adjusted for alcohol use disorder | | | | | | | |  |  | Adjusted for drug use disorder | | | | | | | | |
| --- | --- | --- | --- | --- | --- | --- | --- | --- | --- | --- | --- | --- | --- | --- | --- | --- | --- | --- | --- |
|  | Individuals with mental disorders | | | Unaffected siblings | | |  | |  |  | Individuals with mental disorders | | | Unaffected siblings | | |  | | |
|  | aHR | (CI) | *p* | aHR | (CI) | *p* | RHR | (CI) | *p* |  | aHR | (CI) | *p* | aHR | CI | *p* | RHR | (CI) | *p* |
| Schizophrenia-spectrum disorders | 1.3 | 1.1-1.5 | .003 | 2.2 | 1.7-2.7 | <.001 | 0.6 | 0.5-0.8 | <.001 |  | 1.1 | 0.9-1.3 | .21 | 2.2 | 1.8-2.7 | <.001 | 0.5 | 0.4-0.7 | <.001 |
| Bipolar disorder | 1.6 | 1.2-2.1 | .001 | 1.1 | 0.6-2.1 | .71 | 1.5 | 0.7-2.9 | .28 |  | 1.8 | 1.4-2.4 | <.001 | 1.1 | 0.6-2.0 | .87 | 1.6 | 0.9-3.2 | .14 |
| Depressive disorder | 2.4 | 2.2-2.7 | <.001 | 1.2 | 0.9-1.5 | .17 | 2.0 | 1.5-2.6 | <.001 |  | 2.5 | 2.3-2.8 | <.001 | 1.1 | 0.9-1.4 | .36 | 2.3 | 1.8-2.9 | <.001 |
| Anxiety disorder | 2.1 | 1.9-2.4 | <.001 | 1.5 | 1.2-1.9 | <.001 | 1.4 | 1.1-1.8 | .01 |  | 2.0 | 1.8-2.3 | <.001 | 1.5 | 1.2-1.9 | .002 | 1.3 | 1.0-1.7 | .03 |
| Alcohol use disorder | 7.0 | 6.6-7.5 | <.001 | 1.6 | 1.4-1.9 | <.001 | 4.4 | 3.7-5.2 | <.001 |  | 6.2 | 5.7-6.7 | <.001 | 1.6 | 1.4-2.0 | <.001 | 3.9 | 3.2-4.7 | <.001 |
| Drug use disorder | 6.9 | 6.3-7.5 | <.001 | 2.1 | 1.7-2.5 | <.001 | 3.3 | 2.7-4.1 | <.001 |  | 7.7 | 7.2-8.3 | <.001 | 2.1 | 1.7-2.5 | <.001 | 3.7 | 3.0-4.5 | <.001 |
| ADHD | 5.4 | 4.6-6.4 | <.001 | 2.0 | 1.5-2.7 | <.001 | 2.7 | 1.9-3.8 | <.001 |  | 5.0 | 4.2-6.0 | <.001 | 1.9 | 1.4-2.6 | <.001 | 2.6 | 1.8-3.8 | <.001 |
| Autism | 0.7 | 0.3-1.5 | .32 | 2.1 | 1.2-3.8 | .01 | 0.3 | 0.1-0.9 | .03 |  | 0.6 | 0.3-1.5 | .31 | 2.0 | 1.1-3.7 | <.001 | 0.3 | 0.1-0.8 | .02 |
| Personality disorders | 3.4 | 2.9-4.0 | <.001 | 2.4 | 1.8-3.2 | <.001 | 1.4 | 1.0-2.0 | .04 |  | 3.1 | 2.6-3.6 | <.001 | 2.4 | 1.8-3.1 | .03 | 1.3 | 0.9-1.8 | .11 |

Note. aHRs = adjusted hazard ratios. CI = confidence interval. RHR = ratio of hazard ratios. ADHD = attention deficit hyperactivity disorder. Individuals with an inpatient or outpatient diagnosis of mental disorders and their unaffected siblings were compared with 20 age- and gender- matched general population controls. Apart from adjustments for substance use disorders, aHR analyses were adjusted for family income, single status, and immigrant status. See Table 2 for crude hazard ratios.
