# Supplementary material for: Mental disorders and intimate partner violence perpetrated by men towards women: A Swedish population-based longitudinal study
Source: PLoS Med. 2019 Dec 17;16(12):e1002995. doi: 10.1371/journal.pmed.1002995 (PMC6917212; doi:10.1371/journal.pmed.1002995)
Supplement: Attachment — Submitted filename: Responses to reviewers PMEDICINE-D-19-02530..pdf [file pmed.1002995.s010.pdf]

Requests from the editors

E.1. Did your study have a prospective protocol or analysis plan? Please state this (either way) early in the Methods section.

a) If a prospective analysis plan (from your funding proposal, IRB or other ethics committee submission, study protocol, or other planning document written before analyzing the data) was used in designing the study, please include the relevant prospectively written document with your revised manuscript as a Supporting Information file to be published alongside your study, and cite it in the Methods section. A legend for this file should be included at the end of your manuscript.

b) If no such document exists, please make sure that the Methods section transparently describes when analyses were planned, and when/why any data-driven changes to analyses took place.

c) In either case, changes in the analysis—including those made in response to peer review comments—should be identified as such in the Methods section of the paper, with rationale.

Response: We agree that this is a necessary addition. To clarify, we designed the analytical strategy when we conceived this study. However, we did not write it down in a separate protocol. The strategy remained as is described in the methods with no changes to the exposures, outcomes, or statistical approach used.

In the Methods, we have added: "We designed the analytic strategy when the study was conceived including the exposures (main psychiatric diagnoses), outcome (arrests for IPV) and statistical approach (Cox regression)." Page 10 para 1.

E.2. Thank you for your note that study data are available from "...Karolinska Institute Data Access for researchers who meet the criteria for access to confidential data."

However, PLOS Medicine requires that the de-identified data underlying the specific results in a published article be made available, without restrictions on access, in a public repository or as Supporting Information at the time of article publication, provided it is legal and ethical to do so. Please see the policy at: http://journals.plos.org/plosmedicine/s/data-availability and FAQs at: http://journals.plos.org/plosmedicine/s/data-availability#loc-faqs-for-data-policy

Response: The Public Access to Information and Secrecy Act in Sweden prohibits us from making individual level data publicly available. Researchers who are interested in replicating our work can apply for individual level data at Statistics Sweden: www.scb.se/en/services/guidance-for-researchers-and-universities/.

We have added Data Sharing including above information before the References section. Page 22.

E.3. Abstract: Methods and findings: * Please ensure that all numbers presented in the abstract are present and identical to numbers presented in the main manuscript text. There are typos/missing commas in the numbers of the study participants- please fix/add commas.

Response: We have double checked the numbers of the study participants and now the numbers in the abstract are consistent with those in the main manuscript text. We have also added commas for numbers throughout the article.

E.4. Abstract: Methods and findings: Please quantify the main results (with 95% CIs and p values).

Response: We have added 95% CI and *p* values for the main results in the Methods and findings.

E.5. Abstract: Methods and findings: In the last sentence of the Abstract Methods and Findings section, please describe the main limitation(s) of the study's methodology.

Response: we have now added to the Methods and Findings: "Our findings are applicable to severer forms of IPV perpetration which lead to arrest." Page 3 para 1.

E.6. Author Summary: At this stage, we ask that you include a short, non-technical Author Summary of your research to make findings accessible to a wide audience that includes both scientists and non-scientists. The Author Summary should immediately follow the Abstract in your revised manuscript. This text is subject to editorial change and should be distinct from the scientific abstract. Please see our author guidelines for more information: https://journals.plos.org/plosmedicine/s/revising-your-manuscript#loc-author-summary.

Response: We have now added an Author Summary.

Why was this study done?

Intimate partner violence (IPV) perpetrated by men towards women is a substantial global public health challenge and associated with a wide range of poor outcomes in victims.

One of the risk factors for perpetration is mental disorders, but the nature and strength of the links with these disorders is uncertain.

Previous studies have typically measured the presence of mental disorders and perpetration of IPV at the same time, been based on small numbers, relied on self-report measures of IPV, and have not fully considered confounding factors including shared early environment.

What did the researchers do and find?

We identified men with common psychiatric disorders from a population-based sample and among those with a mental disorder the absolute rate of IPV against women ranged from 0.1% for autism and 2.1% for drug use disorders.

We calculated the relative risk of IPV against women in men with these psych disorders. We also compared risk of perpetrating IPV in men with psych disorders with their siblings without these psychiatric disorders in order to account for familial confounding.

Most of the studied mental disorders were associated with a higher risk of IPV against women. The risk increase was two to seven times compared with the general population, and two to four-fold compared with their unaffected siblings.

Highest absolute rates and relative risks for IPV perpetration were found in men with substance use disorders, and substance use comorbidity was associated with an elevated risk in other mental disorders.

What do these findings mean?

Most common mental disorders are associated with increased risk of IPV against women, which is further elevated when there is comorbidity with substance misuse.

Prevention and intervention programmes should consider prioritising assessment and treatment of IPV perpetration among individuals with psychiatric disorders, including those with alcohol and drug use disorders.

Although the relative risk of IPV against women was higher in men with mental disorders, absolute rates of IPV were low. To reduce IPV against women, other modifiable risk factors need to be addressed.

E.7. Introduction: Please conclude the Introduction with a clear description of the study question or hypothesis. A clear description of the study's main objective(s) is missing.

Response: We have adjusted in the Introduction and now state: "Therefore, the aim of this study is to address these uncertainties in the association between mental disorders and men's IPV against women. To this end, we investigated a…". Page 7 para 4.

E.8. Introduction: Please consider revising the final sentence (comment on largest size and statement of primacy), at least consider qualifying it by including the phrase "to date" in your assertion that this is "the largest epidemiological study" in case this status changes in the future.

Response: We have changed the sentence to: "To our knowledge, this is the largest epidemiological study of IPV perpetrators to date and the first to use sibling comparisons." Page 8 para 1.

E.9. Introduction (and Abstract): Please define the abbreviation "ADHD" at the instance of first use.

Response: We have added the full name: Attention Deficit Hyperactivity Disorder, for ADHD in both Abstract and Introduction.

E.10. Methods and Results: Please provide the actual numbers of events for the outcomes, not just the absolute rates. Specifically, provide the actual numbers associated with the rates of IPV for each population (Table 2 data). It is not clear where the absolute rates of IPV are provided for the population controls. Please specify in the first paragraph of the results section where these results are presented.

Response: We have added the event numbers for the outcomes in individuals with mental disorders and unaffected full siblings. The absolute rates of IPV are provided for the population controls in the first paragraph of the Results: “The absolute rates of IPV perpetrated by men towards women ranged from 0.1% in individuals with autism to 2.1% in those with drug use disorder, from 0.2% to 0.8% among unaffected siblings (Table 2), and from 0.1% to 0.4% in the matched general population controls.” Page 13 para 3.

E.11. Methods and Results: Please provide p values for comparisons of hazard ratios in the text, as well as in tables 2 and 3, and appendixes 1, 3, 4, and 5. Please specify the statistical test used for comparisons.

Methods and Results: Please provide the p values for comparisons between groups. Specifically, in the description of the sensitivity analyses (“These group differences were supported by interaction effects between mental disorders (except for autism) and comorbidity (p’s ≤ .01).”) please specify the p value (unless $p<0.001$) and the statistical test used.

Response: We have added *p* values for comparisons of hazards ratios. The statistical test used has been clarified further in the Methods: “In addition, we also conducted interaction analyses between mental disorders and comorbidity of these three disorders to further examine group differences in the Cox regression model.” Page 12 para 3.

E.12. Methods and Results: Please provide the name(s) of the institutional review board(s) that provided ethical approval.

Methods and Results: Please specify whether informed consent was written or oral, or the conditions permitting the waiver of informed consent.

Response: We have added to the Methods: “The project was approved by the Regional Ethics Review Board in Stockholm, Sweden (2013/5:8), which waived the need for informed consent as anonymized register-based data was used.” Page 8 para 3.

E.13. Methods and Results, and Discussion: In the first paragraph of the results, the number of individuals with depressive disorders is missing a comma. Similarly, a comma is missing from the number of individuals reported in the first paragraph of the discussion. Please edit throughout.

Response: We have added missing commas throughout the manuscript, including figures in the Tables.

E.14. Discussion: Please revise the following sentence: “Furthermore, as the comorbidity of substance use disorders substantially increased the risk of IPV perpetration in all the other mental disorders, including autism which did not show a higher risk when compared to general population controls, these findings could help reducing the stigma around IPV perpetration in mental disorders in general as their higher risk is largely due to substance use disorders.”

Specifically, your study is observational and therefore causality cannot be inferred. Please remove language that implies causality, such as “...as their higher risk is largely due to substance use disorders.” This statement implies causality. Refer to associations instead.

Response: We have deleted causal languages and revised this sentence to: "Furthermore, the comorbidity of substance use disorders was associated with a substantially increased risk of IPV perpetration in all the other mental disorders, including autism which did not show a higher risk when compared to general population controls. These findings could help reducing the stigma around IPV perpetration in mental disorders in general as their risk was ~~is largely due to~~ much lower without comorbidity of substance use disorders." Page 16 para 2.

We have also checked throughout the article and removed language that implies causality in our findings.

E.15. Discussion/Conclusion: Please avoid assertions of primacy ("We report for the first time....") and greatest size. Specifically, please revise the following sentence: "In summary, we examined the link between mental disorders and later IPV using the largest sample of IPV perpetrators and for the first time compared to risks in unaffected siblings to account for genetic and family environmental factors."

Response: We have changed the sentence to: "In summary, we examined the link between mental disorders and later IPV using the largest sample of IPV perpetrators to date and, to our knowledge, for the first time compared to risks in unaffected…" Page 21 para 2.

E.16. Discussion/Conclusion: The statement "...and comorbid substance use disorders increased the risk of IPV against women in all of the other disorders examined…" implies causality. Your study is observational and therefore causality cannot be inferred. Please revise and refer to associations instead.

Response: We have revised the sentence to: "…and comorbid substance use disorders was associated with an increased risk of IPV against women in all of the other disorders examined." Page 21 para 2.

E.17. Table 2, Table 3, Figure 1, Appendices 1, 3, 4, and 5: Please define the abbreviation "CI" in the legend.

Response: We have added a note "CI = confidence interval." as suggested.

E.18. All Tables and Figures: Please define the abbreviation "ADHD" in the legend.

Response: We have defined ADHD (attention deficit hyperactivity disorder) in all Tables and Figures.

E.19. Table 1, and Appendix 2: Please clarify which variables are N (%) and which variables are mean (SD).

Response: We have clarified N (%) and SD on Table 1 and Appendix 2.

E.20. Please ensure that the study is reported according to the STROBE guideline, and include the completed STROBE checklist as Supporting Information. When completing the checklist, please use section and paragraph numbers, rather than page numbers. Please add the following statement, or similar, to the Methods: "This study is reported as per the

Strengthening the Reporting of Observational Studies in Epidemiology (STROBE) guideline (S1 Checklist)."

Response: We have completed STROBE checklist as Supporting Information and added the statement to the Methods. Page 13 para 2.

Reviewer 1

R1.1. In the statistical analyses of the Methods section, it says 'We compared patients' unaffected full siblings with 20 age and gender- matched general population controls with matched conditional logistic regression'. What does this mean and what is it for? We have some odd ratios here but never appeared anywhere in the paper as only Hazard Ratios were applied throughout the paper.

Response: We are grateful to this reviewer for spotting this mistake. We did in fact use Cox regression throughout, which calculates hazard ratios and accounts for time-to-event, as stated in the beginning of the Statistical Analyses.

We have changed the sentence to: "For each patient, up to 20 general population controls without the studied mental disorders were matched by age (birth year) and gender. We adopted Cox regression to control for time-to-event, and account for potential impact of death as a competing event of IPV perpetrated by men towards women." Page 11 para 1. Meanwhile, we have replaced "odds" with "hazard" or "risk" throughout the article.

R1.2. Competing risk. Cox models were applied in the paper to assess the risks, and the outcome is the men's IPV against women other than all-cause mortality, therefore death could be a competing risk in the survival analysis. Can authors elaborate what the death rates are in these cohort? What's its impact on survival analysis in terms of competing risk?

Response: An important point. In our analyses, we did take into account the competing risk of death and should have been more clear about this. Specifically our approach was to use a Cox Regression model that treated 'failures' from the outcome of interest (i.e. IPV) as an event and 'failure' from other causes (death) as censored observations (rather than omitting those who died during the follow up from the analyses).

We have now provided information on rates of death and further specified our Cox regression model in the Methods: "We adopted Cox regression to control for time-to-event, and account for potential impact of death as a competing event of IPV perpetrated by men towards women. In the current study, the rate of death during follow up was higher among men with mental disorders (1.6% to 7.1%) than their matched general population controls (0.4% to 1.1%). Cox regression showed that compared to general population controls, men with mental disorders were 3 to 11 times more likely to die during the follow up. Thus, in our Cox regression, instead of omitting people who died during follow up from the survival analyses, we treated "failure" from death as a censored observation, while "failure" from the outcome of interest (i.e., IPV) as an event." Page 11 para 1.

R1.3. As all the cox models were adjusted for potential confounders, we would like to see the influence/impact of these confounders in the analyses, such as the impact of family income, single status, and immigrant status. We didn't see these confounders presented and discussed in the results or discussion sections.

Response: A helpful comment. All of the aHR (adjusted hazard ratio) were adjusted for potential confounders, including family income, single status, and immigrant status. For the main results, we have provided ranges of hazard ratios for confounders: "HRs ranged from

1.7 to 2.2 for family low-income, 1.2 to 2.1 for single status (expect for in models testing effects of psychosis and bipolar disorder on IPV), and 3.9 to 6.9 for immigrant status across models." Table 2 page 30.

We have also added to the Discussion: "Low income was associated with IPV perpetrated by men towards women. This finding is consistent with existing research on the link between financial distress and increased IPV (41). In addition, individuals who were not married were more likely to commit IPV against women. This could mean that on average marriage implies a more stable and committed relationship than unmarried partnership, and thus is associated with reduced IPV risks. Furthermore, we found that immigrant status (born outside of Sweden) was associated with a higher risk of IPV against women, which may be explained by cultural differences (2)." Page 17 para 3.

Reviewer 2

R2.1. In the discussion it could be addressed that individuals with certain diagnoses, such as autism could by their specific pathology, have less access to victims. Could that be a possible source of bias? This could be of interest to comment.

Response: A very helpful suggestion and we agree. In the Discussion, we have added: "Moreover, it could be that individuals with autism are less likely to have intimate partners and thus have less opportunity for violence against partners. In addition, those who have partners might present with less severe symptoms of autism." Page 17 para 1.

R2.2. The most interesting, and possibly promising finding is the results that substance use disorders and ADHD are related to IPV since, if this is a causal effect, treatment of these disorders could reduce the risk in these groups since both those conditions have evidence based treatments. The notion of the authors that facilities offering treatment to these groups should address IPV and offer parallel treatment, especially since there are upcoming evidence that specific treatment for patients motivated are effective (sorry, upcoming controlled studies from our group).

But, this is probably not, even though relevant, reducing the great prevalence of IPV against women in society. It would be interesting to address how large the proportion of the investigated group is on the total IPV in the population, if possible.

In summary, this paper is well worth publication. Just adding a few comments on generalizability would be at place.

Response: Another helpful comment. We agree that management of IPV could include the parallel treatment of substance disorders and ADHD. We have added to the Discussion:

"Overall, we have shown that mental disorders, particularly substance use disorders, personality disorders, and ADHD, are risk factors of IPV perpetration. Therefore, treatment of these disorders could potentially reduce the risk in these groups, especially as evidence-based treatments exist (42-45). It has been reported that among ADHD patients receiving medication, a significant reduction of criminality rate is observed (46). Furthermore, integrated intervention of mental disorders and IPV may be particularly helpful. This approach is supported by a randomized controlled trial of cognitive behavioural therapy that reduced both symptoms of substance use disorders and partner violence among male offenders (47)." Page 17 para 4.

Regarding the proportion of the investigated group (individuals with mental disorders) to the total IPV in the population, we have calculated the population attributable risk percent (PAR), which is an estimate of the percent of IPV in the total population (individuals with mental disorders and their matched general population controls) that can be attributed to a certain mental disorder. This approach assumes causality, and therefore we have been cautious in its interpretation to explain that it can be interpreted as an estimate of the maximum effect an intervention could have.

We have added to the Discussion: "Assuming causality, population attribution risk percentages can be interpreted as the maximum possible impact that fully treating a disorder

would have on IPV – these ranged from 0.2% for autism to 22.3% for drug use disorders. Treating common deficits such as affect regulation, substance misuse, and specific symptoms of mental disorders might be an important step to prevent IPV against women in some individuals with these disorders. ” Page 18 para 2.

Reviewer 3

R3. If there is one area that is below the generally high quality seen in the overall paper and is in need of further narrative attention, it is the authors' discussion of the etiological links between mental illness and IPV (including the role of substance use in IPV perpetration). There was no discussion of how or why mental illness might be linked to IPV in the Introduction, and the authors' coverage of this topic in the Discussion was severely lacking in breadth and depth. This is an exceedingly important issue in the IPV field in particular, as large sections of the field steadfastly refuse to acknowledge the role of mental illness in any form (e.g., personality traits, specific diagnoses, even alcohol use) as being causally related to IPV perpetration. This resistance, often rooted in protofeminist models of patriarchal socialization, consider such factors as excuses rather than the actual causes of IPV, which are presumed to be rooted in acceptance of personal responsibility. The main point is that there should be no a priori presumption that a large section of readers of this article will accept the very idea of this research, much less the actual findings. Therefore, more careful attention needs to be paid to specific etiological models of how, why, and which mental illnesses are connected to IPV perpetration. While there are a few sentences devoted to some potential mechanisms on p. 14, the authors state that (a) emotion regulation might be involved in emotion disorders (of course); (b) that the psychoactive properties of alcohol might relate to aggression (of course); and (c) that two subtypes of IPV might be important considerations (even though the subtype construct has largely fallen out of scientific favor). The authors are encouraged to be more mindful in their discussion of relevant theory and to more conscientiously discuss the actual and potential mechanisms associated with these models that link mental disorder to IPV perpetration.

Response: Thank you for this helpful comment. We have added more discussion on the etiological links between mental disorders and IPV, both in the Introduction and Discussion and have highlighted the ecological framework used by the WHO and others to conceptualise potential mechanisms.

In the Introduction, apart from existing speculation about the possible reason for perpetration of IPV in people with autism, we have added more discussion on possible explanations for the links with other disorders. We now write: "One potential risk factor for IPV against women is mental illness and etiological links may differ between different disorders. Common deficits in mental disorders such as poor interpersonal skills and emotional dysregulation and specific core symptoms of certain disorders, such as impulsivity manifested in ADHD and substance use disorders, and hostility exhibited in some people with mood disorders and antisocial personality disorder (9-12) have been linked to IPV against women (13, 14)." Page 6 para 2.

In the Discussion, we have rewritten: "Apart from developmental history and current characteristics of individuals, the WHO ecological framework highlights that environmental factors including gender disadvantage (e.g., in education and employment), structural factors and characteristics of the relationship, could also contribute to IPV against women (1, 49, 50)." Page 18 para 2.

We have deleted discussions around the subtypes of IPV: "~~Furthermore, different developmental mechanisms might be underlying different types of perpetration, such as family-only perpetrators and generally violent and antisocial perpetrators.~~ ~~[46,47]~~"

We have added to the Discussion: "Assuming causality, population attribution risk percentages can be interpreted as the maximum possible impact that fully treating a disorder would have on IPV – these ranged from 0.2% for autism to 22.3% for drug use disorders. Treating common deficits such as affect regulation, substance misuse, and specific symptoms of mental disorders might be an important step to prevent IPV against women in some individuals with these disorders. These findings also suggest that to reduce men's IPV against women, other modifiable risk factors in addition to mental disorders need to be considered." Page 18 para 2.

We also have added: "Our findings also highlight the need for examining underlying mechanisms. In addition to providing treatment for common deficits and specific core symptoms of mental disorders, it is important to examine factors at the relational level. It is likely that individuals with mental disorders selectively end up in abusive intimate partnerships, which could lead to reactive violence towards partners. Moreover, there has been evidence of assortative mating (or non-random mating) within and across major mental disorders such as substance use disorders, schizophrenia, depression, and ADHD (51), which might increase the risk of IPV perpetration due to cognitive and social impairments in both partners. Empirical studies are needed to examine potential mediators linking mental disorders to IPV perpetration." Page 18 para 3.
