# Supplementary material for: Mental disorders and intimate partner violence perpetrated by men towards women: A Swedish population-based longitudinal study
Source: PLoS Med. 2019 Dec 17;16(12):e1002995. doi: 10.1371/journal.pmed.1002995 (PMC6917212; doi:10.1371/journal.pmed.1002995)
Supplement: Attachment — Submitted filename: Review PLOS.docx [file pmed.1002995.s009.docx]

This paper address a very important issue from societal perspective. Little is known on what factors are related to severe IPV. The authors correctly address the problems with previous studies such as the imprecision in previous studies. Also, the effect of programs addressing treatment of IPV perpetrators are hampered by many reasons such as selected samples, biased reports, unclear control groups etc.

This study is very well designed by e.g. using sibling comparison. Also, this study utilizes the unique conditions of registers in Sweden, health care databases and the unique crime recording of IPV. The registers of criminality and health care in Sweden provide unique coverage of the total population with extremely low proportion of missing data and generally high quality databases.

The hierarchical assignment of diagnoses used is reasonable and replicate previous studies.

Statistics seem to be appropriate. But, review of this matter should be performed by someone more skilled in statistics than I am.

In the discussion it could be addressed that individuals with certain diagnoses, such as autism could by their specific pathology, have less access to victims. Could that be a possible source of bias? This could be of interest to comment.

The most interesting, and possibly promising finding is the results that substance use disorders and ADHD are related to IPV since, if this is a causal effect, treatment of these disorders could reduce the risk in these groups since both those conditions have evidence based treatments. The notion of the authors that facilities offering treatment to these groups should address IPV and offer parallel treatment, especially since there are upcoming evidence that specific treatment for patients motivated are effective (sorry, upcoming controlled studies from our group).

But, this is probably not, even though relevant, reducing the great prevalence of IPV against women in society. It would be interesting to address how large the proportion of the investigated group is on the total IPV in the population, if possible.

In summary, this paper is well worth publication. Just adding a few comments on generalizability would be at place.
