# Supplementary material for: Mental disorders and intimate partner violence perpetrated by men towards women: A Swedish population-based longitudinal study
Source: PLoS Med. 2019 Dec 17;16(12):e1002995. doi: 10.1371/journal.pmed.1002995 (PMC6917212; doi:10.1371/journal.pmed.1002995)
Supplement: Attachment — Submitted filename: R3 Revision letter PMEDICINE-D-19-02530..docx [file pmed.1002995.s011.docx]

**Editorial Requests**

FIGURES and GRAPHICS:

1. Please ensure that all main figures are uploaded in .tif or .eps format. For more information about how to convert your figure files, please see our guidelines: <http://journals.plos.org/plosmedicine/s/figures>.

2. Please remove embedded figures from your manuscript file and ensure the most up to date versions of your figures are uploaded to our submission system as separate TIF or EPS files. Please do not remove the figure legends and captions.

3. Please ensure that all main figure files are cited in ascending numerical order in the main text of the article.

4. Please ensure that all main figures are referenced in the manuscript as Fig 1, Fig 2, etc. (including capitalization), rather than Figure 1, Figure 2, etc. Please note, however, that the file names themselves must not include the space, i.e. Fig1.tif, Fig2.tif, etc.

5. Please ensure that all main figure legends are placed after the paragraph containing their first in-text citation.

**Response:** We have now uploaded Fig 1 in .tif format with a resolution of 600dpi and removed embedded figure in Fig 1 from the manuscript file. We have moved the figure legends to page 16, right after the paragraph containing its first in-text citation.

TABLES and BOXES:

1. Please cite main tables in ascending numerical order in your main article file.

2. Please ensure that all main tables in their entirety (including titles and footnotes), are placed after the paragraph containing their first in-text citation.

3. Tables 1, 2 and 3 use returns to control text wrapping. This is indicated by a [¶ or ↵] symbol when "Show Paragraph Marks" is turned on in Word. Please either split those cells into separate cells to achieve alignment or remove the returns.

**Response:** All tables are now cited in ascending numerical order in the main article and we have moved Tables 1, 2, and 3 right after its first in-text citation. We have removed the “returns” in Tables as suggested.

METADATA:

1. Please ensure that the CRediT author contributions listed for each co-author are completed in full. To qualify for authorship, each author must meet at least one of the seven core contributions (conceptualization; data curation; formal analysis; investigation; methodology; software; validation), as well as at least one of the writing contributions (original draft preparation; review and editing). Authors may also be noted against any of the remaining contributions, however these alone will not qualify a contributor for authorship.

Authors Yasmina Molero and Louise M Howard are noted as contributing to "Writing - review and editing" only, which, as mentioned above, does not qualify them for authorship. Please ensure that the full contributions of each author are acknowledged in the "Add/Edit/Remove Authors" section of our submission form. If they do not meet criteria for authorship please let us know as soon as possible by emailing [plosmedicine@plos.org](mailto:plosmedicine@plos.org).

**Response:** In addition to “writing”, author Yasmina Molero has contributed to Investigation and author Louise M Howard has helped in the study design (Methodology). Therefore, they are qualified as authors. We have added these contributions for these two authors in the submission system.

2. Please accurately complete the 'Competing Interests' section on our submission form, including any COIs declared by your co-authors. If you have no competing interests to declare, please add "The authors have declared that no competing interests exist". Otherwise please declare all competing interests beginning with the statement "I have read the journal's policy and the authors of this manuscript have the following competing interests:"

3. Your co-author, Dr. Nevado, has declared the following competing interest in relation to this manuscript. Please include it in the main competing interest statement in our system, sticking as closely as possible to Dr. Nevado's wording, using only their initials. "Some members of the laboratory led by Alejo Nevado-Holgado are funded by Johnson and Johnson. - The laboratory of Alejo Nevado-Holgado received funds from Ono Pharma in 2018."

Your co-author, Dr. Larsson, has declared the following competing interest in relation to this manuscript. Please include it in the main competing interest statement in our system, sticking as closely as possible to Dr. Larsson's wording, using only their initials. "H Larsson has served as a speaker for Evolan Pharma and Shire and has received research grants from Shire; all outside the submitted work."

Your co-author, Dr. Howard, has declared the following competing interest in relation to this manuscript. Please include it in the main competing interest statement in our system, sticking as closely as possible to Dr. Howard's wording, using only their initials. "I am lead investigator on a UK Research and Innovation funded Mental Health Network on Violence, Abuse and Mental Health."

4. Please ensure only the authors' initials are included in the competing interest statement, not their full names or titles.

**Response to 2-4:** We have added detailed completing interest for Dr. Nevado, Dr. Larsson, and Dr. Howard to the submission system. We have used initials of the authors.

5. Please provide a detailed Financial Disclosure statement on the submission form in full sentences, and include URLs, the initials of the receiving author, and specific grant numbers wherever possible.

**Response:** This has been added to the submission system.

6. In accordance with PLOS' data policy, please ensure that your Data Availability Statement in the submission form clearly identifies how readers can access your data. Data cannot be available on request, or only accessible by contacting one of the authors. Note that your Data Availability Statement will be typeset as it is written, so please ensure it is in complete sentences and appears as you would like it to in the published version.

For details on our data policy, please see <http://journals.plos.org/plosmedicine/s/data-availability>

**Response:** We have double checked the Data Availability Statement and added them to the submission system.

ABSTRACT and SUMMARY POINTS:

1. Please provide between 3 and 5 summary points of your article which summarise the key points. Please ensure these are at the beginning of your article file, after the title page in bullet point rather than parargraph format.

**Response:** The Author Summary in bullet points has been added.

ARTICLE FILE:

1. Please indicate the corresponding author in the byline by placing an asterisk (*) after their affiliation number.

**Response:** This has been added as requested.

2. Please include the corresponding author's email address on the title page of your manuscript, indicated by an asterisk (*). Only an asterisk and the email address itself are required.

**Response:** This has been added.

3. Please remove author degree titles from the byline of your article file.

**Response:** Author degree titles have been removed as required.

4. Please ensure that all authors have at least one affiliation listed on the title page, and that all listed affiliations are attributed to at least one author. Affiliations should be listed in ascending numerical order in both the affiliation list and in the author byline.

**Response:** This has been adjusted as suggested.

5. Please remove the funding information from your manuscript file. The published version is pulled automatically from the “Financial Disclosure” field in the submission form and must not be duplicated. Please therefore take this opportunity to check that the information in this field is correct and completed in full.

**Response:** We have removed the funding information from manuscript.

6. Please remove the data availability information from your manuscript file as this is automatically pulled from the "Data Availability" field in the submission form and must not be duplicated. Please therefore take this opportunity to check that the information in this field is correct and completed in full.

**Response:** We have removed the “Data Availability” field from the manuscript.

7. Please ensure that your Ethics statement is available in the Methods section of your manuscript in its entirety.

**Response:** The Ethic statement is available in the Methods.

REFERENCES:

1. Please put all reference citations in square brackets separated by either commas or dashes with no spaces, e.g. [1,2,3] or [1-3].

2. Please make sure all references are cited in ascending numerical order in the text.

**Response:** All references are cited in ascending numerical order in the text and we have now used square brackets.

SUPPORTING INFORMATION:

1. Please ensure that all Supporting Information files use the following format verbatim throughout the manuscript (including legends and all in-text citations): S1 Fig, S1 Table, S1 Text, S1 Data, etc. Each file type should start at 1 and increase numerically. Please note, however, that the file names themselves must use an underscore rather than a space, i.e. S1_Fig.tif, S1_Table.xlsx, S1_Text.doc, S1_Data.xlsx, S1_STROBE_Checklist.docx, etc.

2. Please ensure that each supporting information file has a legend at the end of the manuscript file, after the Reference list.

3. Please note that your submission cannot contain any references to generic "supporting information" files and instead must refer to specific files using our naming convention (S1 Fig, S1 Table, S1 Text, S1 Data, SI PRISMA Checklist, etc.)

**Response:** We have cited Supporting Information as advised. All supporting Tables are currently placed in the end of the manuscript.

Editorial Requests:

Abstract: Background: In the final sentence “In a longitudinal design, we examined the association between mental disorders and IPV perpetrated by men towards women in a population-based sample and used sibling comparisons to control for all factors that make siblings similar, such as genetic and early family environmental factors.” please remove the word “all” from “control for all factors”. The text has been changed to strikethrough but not removed.

Abstract: Methods and findings: Please remove the final “in men” from the sentence: “We matched individuals by age and gender to general population controls (ranging from 186,017 to 1,719,318 controls), and calculated the hazard ratios of IPV against women in men.” because you have already explained you are studying men.

Abstract: Methods and findings: Please add the word “studied” to the sentence: In comparison to general population controls, all psychiatric diagnoses except autism were associated with an increased risk of IPV against women in men, with hazard ratios (HR) ranging from 1.5 (95% CI, 1.3-1.7) to 7.7 (7.2-8.3), ps < .001.” so that it reads “...all psychiatric diagnoses studied except autism…”.

Abstract: Methods and findings: Please change “had higher risk” to “had a higher risk” in the following sentence: “In sibling analyses, we found that men with depressive disorder, anxiety disorder, alcohol use disorder, drug use disorder, ADHD (Attention Deficit Hyperactivity Disorder), and personality disorders had higher risk of IPV against women than their unaffected siblings, with ratio of hazard ratios (RHRs) ranging from 1.7 (1.3-2.1) to 4.4 (3.7-5.2), ps < .001.”

Abstract: Methods and findings: Please change “folds” to “fold” in the sentence: “Sensitivity analyses showed three to five folds higher risk of IPV against women in men when comorbid substance use disorders and personality disorders were present, compared to risk when these comorbidities were absent.”

Abstract: Methods and findings: Please change “were” to “was” in the sentence: “In addition, increased IPV risk were also found in those without a previous IPV.”

Abstract: Methods and findings: Please revise the final sentence to: “A limitation of our study is that our analyses were restricted to IPV leading to arrest, suggesting that these results may be applicable to more severe forms of IPV perpetration.” or “Our analyses were restricted to IPV leading to arrest, suggesting that the applicability of our results may be limited to more severe forms of IPV perpetration.” to make the main limitations of the study clear to the reader.

Abstract: Conclusions: Please revise the final sentence to: “The findings support the development of IPV risk identification and prevention services among men with substance use disorders as an approach to reduce the prevalence of IPV.” or some similar clarification on what is meant by “development of services”.

Author Summary: Why was this study done?: Please remove the words “substantial” and “wide” from the first bullet point “Intimate partner violence (IPV) perpetrated by men towards women is a substantial global public health challenge and associated with a wide range of poor outcomes in victims.” These were changed to strikethrough text but not removed.

Author summary: What did the researchers do and find?: Please revise the first point to: “We identified men with common psychiatric disorders from a population-based sample, and compared their risk of IPV against women with age-matched general population controls, and also with their unaffected siblings to account for familial confounding.”

Introduction: In the second paragraph, please revise “majority” to “a majority” and “been with” to “been done with” in the following sentence to read: “However, a majority of existing empirical studies have been done with small sample sizes, often based on selected samples, relied on self-report of risk factors and outcomes, and most importantly lacked adequate adjustment of confounders such as familial factors.”

**Response:** We have made all of the above editorial changes as advised.

Introduction: In the fourth paragraph, please delete the sentence “Clarifying whether these associations reflect causal effects or confounding can assist in developing more effective prevention and intervention programs (25).” to avoid any misconceptions as your study cannot address causality.

**Response:** We have changed the sentence to: “Clarifying whether these associations reflect causal effects or confounding can assist in developing more effective prevention and intervention programs (25).” Page 7 para 3.

Introduction: Please the revise the last sentence of the fourth paragraph to avoid misleading readers to the conclusion that causality can be inferred from your observational study, as well as to avoid any misunderstanding that the mental disorders you are studying can be considered modifiable factors contributing to IPV: “To date, many intervention and prevention programs targeting perpetrators of IPV typically have limited effectiveness (26), and this may be partly related to the lack of identified IPV-associated factors in these programmes.”

**Response:** We have changed this sentence to: “To date, many intervention and prevention programs targeting perpetrators of IPV typically have limited effectiveness [26], and this may be partly related to the lack of modifiable factors in these programmes.” Page 7 para 3.

Methods: Statistical Analyses: Please specify in this sentence that the siblings were gender-matched to the patients: “To account for possible familial confounding, we conducted additional analyses with unaffected, gender-matched, full siblings of patients as controls.”

Methods: Statistical Analyses: Please revise the following sentence to change “arrest of general domestic violence” to “arrest for general domestic violence”: “Fourth, we examined the association between mental disorders and arrest of general domestic violence to examine whether mental disorders were associated with IPV against women and with general domestic violence in a similar pattern in men.”

Results: Sensitivity Analyses: Please change “were” to “was” in the following sentence: “We found that the hazard ratio of IPV against women in psychiatric disorders were increased with comorbid substance use disorders and personality disorders (except in autism).”

Results: Please spell out the abbreviation for “ROR” at its first use in this sentence: “Comparisons with hazard ratios in unaffected full siblings (vs general comparison) also showed similar patterns, with RORs ranging from 1.4 (1.1-1.8) to 3.7 (3.0-4.4), ps < .001.”

Disucssion: Please change “drug” to “drugs” in the following sentence: “Individuals with psychiatric disorders are also likely to use alcohol and drug as coping strategies to deal with difficult symptoms associated with their illnesses (39, 40).”

Discussion: Please change “reducing” to “reduce” and “their risk” to “IPV risk” in the following sentence: “These findings could help reducing the stigma around IPV perpetration in mental disorders in general as their risk was much lower without comorbidity of substance use disorders.”

Discussion: Please change “control” to “controls” in the following sentence: “We found that schizophrenia-spectrum disorders showed higher risk of IPV perpetration than general population control.”


Discussion: Please add “with symptoms” to the following sentence so that it reads: “In addition, research which showed associations between psychosis and domestic homicides also reported that perpetrators with symptoms at the time of offense were less likely than perpetrators without symptoms to have previous violence convictions (44)

Discussion: Please change “partner violence” to “intimate partner violence” in the following sentence: “This approach is supported by a randomized controlled trial of cognitive behavioural therapy that reduced both symptoms of substance use disorders and partner violence among male offenders (52).”

Discussion: Please change “benefit” to “benefits the” in the sentence: “Second, we tested the associations between mental disorders and IPV perpetration in a population-based sample, which benefit generalisability of the findings.”

Discussion: Please change “severer forms of IPV” to “more severe forms of IPV” in the following sentence: “As it has been widely recognized that victims of partner abuse tend to not report the abuse to the police and many individuals might have already perpetrated IPV prior to the age of 15 (the age of inclusion in the Crime register), IPV was under-reported (60) and our findings are specific to severer forms of IPV perpetration which lead to arrest and likely cause significant and negative consequences for victims, severe morbidity and in rare cases mortality.”

Conclusion: Please change “was associated” to “were associated” in the following sentence: “Substance use disorders as a primary diagnosis had the highest risk of IPV among studied mental disorders and comorbid substance use disorders was associated with an increased risk of IPV against women in all of the other disorders examined.”

Figure 1: The title should be “ratio of hazard ratios”.

Appendix 1: The title should read (HRs) rather than (aHRs).

STROBE Checklist: Other Analyses: “Appendix” is misspelled.

**Response:** We have made these changes as suggested.
